# Self-Administration of JWH-018 A Synthetic Cannabinoid in Experimentally Naïve Rats

**DOI:** 10.4172/2329-6488.1000e128

**Published:** 2015-11-18

**Authors:** Takato Hiranita

**Affiliations:** Division of Neurotoxicology, National Center for Toxicological Research (NCTR), U.S. Food and Drug Administration (FDA), USA

## Editorial

**Figure 1:**
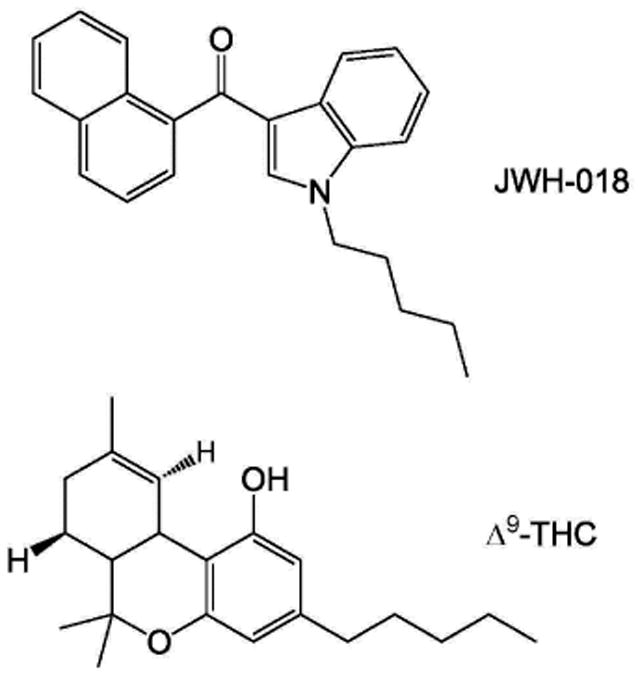
Chemical structures of JWH-018 [1-pentyl-3-(1-naphthoyl)indole] and Δ9-THC [(−)-trans-Δ9-tetrahydrocannabinol].

A recent study by Dr. Maria Antonietta De Luca demonstrated intravenous (IV) self-administration responding (nose-poking) for the synthetic cannabinoid JWH-018 [1-pentyl-3-(1-naphthoyl)indole] (Figure 1) in an experimentally naïve, adult rat species [1]. This finding is unexpected since the phytocannabinoid (−)-trans-Δ9-tetrahydrocannabinol (Δ9-THC, Figure 1), a primary psychoactive constituent in marijuana, has been reported to not maintain IV self-administration responding above vehicle levels in rats [2,3] and rhesus monkeys [4-6]. IV self-administration of synthetic cannabinoids is not unprecedented since several synthetic cannabinoids have been found to maintain IV self-administration responding in experimentally naïve rats [1,7-10], and mice [11-14]. However, the finding by Dr. De Luca is important because JWH-018 has been frequently found in K2/Spice preparations [15-17] and there continues to be an increase in the abuse and non-medical use of various synthetic cannabinoids worldwide [15-17]. Further, the use of marijuana has been recently legalized in two states of the U.S.

The finding by Dr. De Luca is unexpected since response-dependent changes in visual stimuli were not presented at the time when the compound was self-injected. Self-administration of synthetic cannabinoids in most studies has been demonstrated in the presence of response-dependent changes in visual stimuli [3,7-9,11,12,14]. The Dr. De Luca's finding is important because the dopamine D2-like agonist, quinpirole, was not self-administered above vehicle levels in experimentally naïve rats even when a response-dependent injection-paired visual stimulus was presented [18,19]. Further, (-)-nicotine was not self-administered above vehicle levels in experimentally naïve rats in the absence of an injection-paired visual stimulus [20]. In addition, the rate of acquisition of self-administration reported by Dr. De Luca is also unexpected: 100% of fourteen rats assessed [1]. To put this in context, maximal self-administration acquisition rates of the synthetic cannabinoid WIN 55,212-2 using drug naïve, adult rats were reported to be 85.7 % (12 out of 14) at 0.0125 mg/kg/injection [7] or 60.0% (3 out of 5) at 0.01 mg/kg/injection [3]. Finally, the finding by Dr. De Luca stands in marked contrast to the reinforcing effects of Δ9-THC. Despite the demonstrated effectiveness of Δ9-THC as a positive reinforcer in experimentally naïve squirrel monkeys [21], Δ9-THC has been reported to fail to maintain IV self-administration responding above vehicle levels in rats [2,3] and rhesus monkeys [4-6]. Thus, it appears that JWH-018 is a more effective positive reinforcer in rats than Δ9-THC.

As mentioned above, the abuse of synthetic cannabinoids is increasing [15,16]. Despite the low effectiveness of the phytocannabinoid Δ9-THC as a positive reinforcer in a rat species [2,3], Dr. De Luca found a relatively high capacity of the synthetic cannabinoid JWH-018 to induce self-administration responding above vehicle levels in experimentally naïve rats [1]. Unexpectedly, Dr. De Luca also demonstrated self-administration of the endocannabinoid 2-arachidonoylglycerol in experimentally naïve rats [22]. These findings suggest that rats will be a useful model for the further assessment of the abuse potential of various synthetic cannabinoids.
